# Supplementary material for: Chromothripsis during telomere crisis is independent of NHEJ, and consistent with a replicative origin
Source: Genome Res. 2019 May;29(5):737–49. doi: 10.1101/gr.240705.118 (PMC6499312; doi:10.1101/gr.240705.118)
Supplement: Supplemental Material [file supp_gr.240705.118_Supplemental_file_1.zip › contigs/annotated_contigs/DB79/contig.3.DB79_length_391_mean_cov_8.82352941176.docx]

**DB79_length_391_mean_cov_8.82352941176**

```
ATTTTCCTTTCTCCTGCTGGTTTTGGTAAACAGGTTATGCTAGACAAATTCATAAGTGAGTTGAGGCTGGCAGTTGGTGACAGACATTT
 >chr1:180282366-180282623 + E=2e-143
CAACCTCAATTATTAGGCTGACATATGTAGGTAGTATAATAGCTAGTCCAATAGGTTAAGACTCCTAGATAGCACAGCGAGGTTTCTCC

AGCATTATCCCTCCAGAATGATTTGGCTTTTGTTTCCGCCAAGCATCCCAGAAACACTATCAGCTTGAAATACCTTAGT|ATTTCA|AT
                                                                                      >
ATACATATAATTTACATATAATATACATATA|ATT|TACATATAATTTACCATCTTTACCATTTTACCATTTTTAAGTGTACAGTGTTT
chr15:47369104-47369137 - E=2e-02 p=0e+00  >chr1:180196685-180196748 + E=5e-18
ATGCTTTTTTTAAGAAAA|AACCAATTACTGAAGTGCTCT
```
